# Comparison of infant bone marrow- and umbilical cord-derived mesenchymal stem cells in multilineage differentiation

**DOI:** 10.1016/j.reth.2024.09.011

**Published:** 2024-10-03

**Authors:** Szu-Hsien Wu, Jin-Huei Yu, Yu-Ting Liao, Po-Hsin Chou, Ming-Hsuan Wen, Kuang-Kai Hsueh, Jung-Pan Wang

**Affiliations:** a Department of Surgery, School of Medicine, National Yang Ming Chiao Tung University, Taipei, Taiwan; b Division of Plastic and Reconstructive Surgery, Department of Surgery, Taipei Veterans General Hospital, Taipei, Taiwan; c Division of Plastic and Reconstructive Surgery, Department of Surgery, School of Medicine, National Defense Medical Center, Taipei, Taiwan; d Department of Orthopedic Surgery, Taoyuan General Hospital, Ministry of Health and Welfare, Taoyuan, Taiwan; e Department of Orthopaedics & Traumatology, Taipei Veterans General Hospital, Taipei, Taiwan

**Keywords:** Infant bone marrow-derived stem cells (Infant BMSCs), Umbilical cord-derived mesenchymal stem cells (UCSCs), Multilineage differentiation

## Abstract

We compared infant bone marrow-derived mesenchymal stem cells (infant BMSCs) with umbilical cord-derived mesenchymal stem cells (UCSCs) by assessing multilineage differentiation. Proliferation was gauged through changes in cell numbers and doubling time. Senescence-related genes (*p16*, *p21*, and *p53*), senescence-associated β-galactosidase (SA-β-gal), and γH2AX immunofluorescence determined senescence presence. Superoxide dismutases (SODs) and genes related to various differentiations were analyzed using reverse transcription-quantitative polymerase chain reaction (RT-qPCR). Differentiation was confirmed through histochemical, immunohistochemical, and immunofluorescence staining. Infant BMSCs surpassed UCSCs in proliferation. Infant BMSCs exhibited lower senescence-related gene expression at late passages, upregulated antioxidant enzymes during early passages, and reduced SA-β-gal staining. Chondrogenic gene expression (*SOX9*, *COL2*, and *COL10*) was enhanced in infant BMSCs, along with improved immunohistochemical staining. Infant BMSCs showed higher expression of osteogenic (*ALP* and *OCN*) and adipogenic (*PPARγ* and *LPL*) genes, confirmed by histochemical staining. However, UCSCs had higher expression of tenogenic genes (*MMP3*, *SCX*, *DCN*, and *TNC*). Hepatogenic differentiation potential was similar, with no significant difference in hepatogenic gene expression (*ALB* and *TAT*). Compared to UCSCs, infant BMSCs demonstrated superior proliferation, reduced senescence, increased antioxidant capacity, and enhanced differentiation potential toward chondrogenic, osteogenic, and adipogenic lineages.

## 1 Introduction

Stem cell therapy is emerging as a novel treatment for incurable diseases via replacing defective cells with stem cells in regenerative medicine. As popular stem cell resources, mesenchymal stem cells (MSCs) can be obtained from a variety of tissues such as bone marrow, fat, amnion [1], and umbilical cord [2]. MSCs derived from bone marrow (BMSCs), adipose tissue (ADSCs), and umbilical cord (UCSCs) applied to therapies for different diseases have been studied many times and developed for clinical application [3]. There are many studies to compare the proliferation and potential of different tissue-derived MSCs. Compared to BMSCs and ADSCs, UCSCs showed the most prolonged culture period, highest proliferation potential, and the capacity to differentiate into osteogenic lineage [[4], [5], [6]].

UCSCs are derived from the tissue surrounding the blood vessels of the umbilical cord and are categorized as mesenchymal stem cells [7]. UCSCs have a relatively short doubling time, and their collection is not subject to ethical constraints [8]. Additionally, the utilization of UCSCs is not restricted by medical-legal regulations [6]. In many instances, UCSCs are obtained from umbilical cords, which are typically regarded as medical waste [9].

The infant BMSCs can be collected from the phalanges of excised redundant fingers or compared to the thumb of polydactyly children under two years old by reconstructive surgery. They showed higher proliferation and differentiation potential compared with adult ones [10]. The collecting process of the infant BMSCs from polydactyly children will not cause extra pain to patients because the BMSCs could be isolated from the abandoned phalanges or carpal bones, which is considered surgical waste.

The proliferation and differentiation potential of infant BMSCs have not been previously compared with UCSCs. However, we have previously compared infant ADSCs and BMSCs, both of which were isolated from excised polydactyly fat tissue and phalanges typically discarded as surgical waste. Despite UCSCs being considered among the youngest samples in most MSCs, we found that infant ADSCs exhibited a higher differentiation potential than UCSCs. In this study, our objective is to examine and compare the proliferation and differentiation potential of infant BMSCs and UCSCs, aiming to gain insights into the stemness and potential applications of these two types of MSCs.

## 2 Materials and methods

### 2.1 Cell culture of UCSCs and infant BMSCs

We obtained UCSC cell lines (ATCC PCS-500-010) comprising UCSC-1 (lot #64310874, passage 1), UCSC-2 (lot #70005074, passage 2), and UCSC-3 (lot #70014369, passage 2) from the American Type Culture Collection (ATCC, VA, USA). The characterization of UCSCs was provided in the Certificate of Analysis from ATCC. UCSC-3, used as a representative sample, was further validated by flow cytometry. The results demonstrated positive expression for CD29, CD44, CD73, CD90, and CD105, and negative expression for CD11b, CD14, CD19, CD34, CD45, CD79a, and HLA-DR (Supplementary Fig. S1). These cell lines were maintained in Dulbecco's Modified Eagle Medium (DMEM; Gibco, CA, USA) with low glucose (LG-DMEM), supplemented with 10 % fetal bovine serum (FBS, Hyclone, UT, USA), and antibiotic-antimycotic solution (Corning life science, NY, USA). The culture medium was refreshed every 48 h, and when the cells reached confluence, they were subcultured at a 1:5 split ratio every five to seven days. The cells were incubated at 37 °C in a 5 % CO_2_ environment.

Human infant BMSCs were collected from the bone of surgically excised redundant thumbs during polydactyly reconstruction surgeries in children. The research procedures were carried out in accordance with the guidelines and regulations established by the Institutional Review Board (IRB). Mononuclear BMSCs were isolated using a density gradient centrifugation method. The isolated BMSCs were further confirmed for their ability to adhere to plastic substrates. Flow cytometry analysis verified the cell-surface protein profiles of infant BMSCs, which demonstrated positive expression for CD29, CD44, CD73, CD90, and CD105, while they tested negative for CD11b, CD14, CD19, CD34, CD45, CD79a, and HLA-DR. The results from the flow cytometry analysis have been included in the previous manuscript [10], and a representative example is shown in Supplementary Fig. S1. Nucleated cells were plated at clonal density and cultured in a modified Minimum Essential Medium (α-MEM, Invitrogen, CA) containing 10 % FBS, 100 U/ml penicillin (Invitrogen), 100 μg/ml streptomycin (Invitrogen), and 250 ng/ml amphotericin B (Invitrogen). The growth medium was replaced every 48 h, and the cells were subcultured every four days at a 1:5 split ratio, before reaching 80 % confluence.

### 2.2 Proliferation

To quantify the doubling time and characterize proliferation, both Infant BMSCs and UCSCs were cultured independently in α-MEM or DMEM, each containing 10 % FBS, 100 U/ml penicillin, 100 μg/ml streptomycin, and 250 ng/ml amphotericin B. The culture medium was refreshed every 3–4 days. Consistently, cells were seeded at a density of 1–2 × 10^5^ cells in 10-cm dishes. Subsequently, every 7 days, the cells were recovered and passaged. At each passage, the cell numbers were counted in triplicate cultures. This information was then used to calculate the fold change in cell number and determine the population doubling time, as described in a previous study [11].

### 2.3 RT-QPCR analysis

Cellular RNA was extracted using Trizol reagent (Invitrogen). First-strand complementary DNA (cDNA) was synthesized using random sequence primers and the iScript™ cDNA Synthesis Kit from Bio-Rad® (Hercules, CA, USA). Real-time PCR was conducted to amplify the cDNA in a reaction mixture containing specific primer pairs (refer to Table 1) and Fast SYBR® Green 189 Master Mix from Applied Biosystems (CA, USA). Glyceraldehyde-3-phosphate dehydrogenase (*GAPDH*) was employed as the internal control. The analysis of the results was performed using the software provided with the PCR machine, utilizing the comparative CT (ΔΔCT) method [11].

**Table 1:** Primer sequences used for RT-qPCR analysis.

Gene	Forward primer (5′-3′)	Reverse primer (5′-3′)
*p16*	ATCATCAGTCACCGAAGG	TCAAGAGAAGCCAGTAACC
*p21*	CATCTTCTGCCTTAGTCTCA	CACTCTTAGGAACCTCTCATT
*p53*	CGGACGATATTGAACAATGG	GGAAGGGACAGAAGATGAC
*TERT*	AAATGCGGCCCCTGTTTCT	CAGTGCGTCTTGAGGAGCA
*SOD1*	GTGATTGGGATTGCGCAGTA	TGGTTTGAGGGTAGCAGATGAGT
*SOD2*	TTAACGCGCAGATCATGCA	GGTGGCGTTGAGATTGTTCA
*SOD3*	CATGCAATCTGCAGGGTACAA	AGAACCAAGCCGGTGATCTG
*SOX9*	CCAGGGCACCGGCCTCTACT	TTCCCAGTGCTGGGGGCTGT
*COL2*	TTCAGCTATGGAGATGACAATC	AGAGTCCTAGAGTGACTGAG
*COL10*	CAAGGCACCATCTCCAGGAA	AAAGGGTATTTGTGGCAGCATATT
*ALP*	ACCATTCCCACGTCTTCACATTTG	AGACATTCTCTCGTTCACCGCC
*OCN*	ACCCCAGTTCTGCTCCTCTCCA	TGGGAGCAGCTGGGATGATG
*MMP3*	CTGTTGATTCTGCTGTTGAG	AAGTCTCCATGTTCTCTAACTG
*SCX*	CAGCGGCACACGGCGAAC	CGTTGCCCAGGTGCGAGATG
*DCN*	CTCTGCTGTTGACAATGGCTCTCT	TGGATGGCTGTATCTCCCAGTACT
*TNC*	CCACAATGGCAGATCCTTCT	GTTAACGCCCTGACTGTGGT
*PPARγ*	TCAGGTTTGGGCGGATGC	TCAGCGGGAAGGACTTTATGTATG
*LPL*	TGTAGATTCGCCCAGTTTCAGC	AAGTCAGAGCCAAAAGAAGCAGC
*TAT*	TCAGTTTCCCGTATGCCACC	ATCTTTGGGGGCTTGGATGG
*ALB*	TGCTTGAATGTGCTGATGACAGGG	AAGGCAAGTCAGCAGGCATCTCATC
*GADPH*	ATATTGTTGCCATCAATGACC	GATGGCATGGACTGTGGTCATG

### 2.4 Senescence-associated β-galactosidase (SA-β-gal) assays

To assess cellular senescence in infant BMSCs and UCSCs, we employed a senescence-associated β-galactosidase cell staining kit from Cell Signaling Technology (MA, USA). Cells at passages 8–9 were plated in 6-cm dishes at a density of 1 × 10^5^ cells per dish and incubated for five days at 37 °C in a 5 % CO_2_ environment. Following the manufacturer's guidelines, senescent cells were identified by their blue staining and were observed using a microscope.

### 2.5 γH2AX immunostaining

Infant BMSCs and UCSCs at passage 8–9 were subjected to a series of procedures. First, they were permeabilized using a permeabilization buffer consisting of 0.1 % Triton X-100 in PBS. Subsequently, the cells were fixed with 4 % paraformaldehyde. Following fixation, primary antibodies targeting the phosphorylation of histone variant γ-H2AX (Taiclone Biotech Corp., Taipei, Taiwan) were applied at an appropriate dilution. To visualize the staining, DyLight 488-conjugated goat anti-rabbit IgG secondary antibodies (GeneTex, CA, USA) were used, resulting in green fluorescence. To highlight the cell nuclei, 4,6-diamidino-2-phenylindole (DAPI, Sigma-Aldrich, MO, USA) was employed. Fluorescence intensity was assessed in 60–100 cells using Image-Pro Plus software (version 4.5.0.29, Media Cybernetics, MD, USA). The obtained measurements were then normalized with respect to cell numbers and expressed in arbitrary units.

### 2.6 Chondrogenic differentiation

Infant BMSCs or UCSCs at passages 3 to 5 were subjected to chondrogenic differentiation by placing them in a specialized chondrogenic induction medium (CIM). This medium consisted of serum-free, high-glucose (4.5 g/l) DMEM (HG-DMEM) from Gibco (NY, USA), supplemented with 50 mg/ml ITS plus Premix (BD Biosciences, CA, USA), which included 6.25 mg/ml insulin, 6.25 mg/ml transferrin, 6.25 mg/ml selenious acid, 1.25 mg/ml bovine serum albumin (BSA), and 5.35 mg/ml linoleic acid. To facilitate chondrogenic differentiation, additional components were included, such as 10-7 M dexamethasone (Sigma-Aldrich), 50 mg/ml ascorbate-2-phosphate (Sigma-Aldrich), and 10 ng/ml TGF-β1 (R&D Systems, MN, USA). The cells were cultured under standard conditions at 37 °C with 5 % CO_2_. The medium was refreshed every three days, and the cell pellets were observed and collected on day 21 of the induction process [12].

### 2.7 Immunohistochemistry staining

Immunohistochemistry staining was performed on the chondrogenic pellets formed by infant BMSCs and UCSCs, and the equivalent diameter of these pellets was measured on day 21. The pellets were sectioned and prepared for paraffin embedding. These sections underwent a series of staining procedures, starting with Alcian blue staining (ScyTek Laboratories, UT, USA) to highlight proteoglycans. Subsequently, they were counterstained with nuclear fast red (ScyTek). For the detection of collagen type 2 (COL2) and collagen type 10 (COL10), the sections were deparaffinized, hydrated, treated with 0.4 mg/ml proteinase K for 15 min, and endogenous peroxidase activity was blocked using 3 % hydrogen peroxide (Sigma-Aldrich). After thorough washing and blocking, the sections were incubated with primary antibodies against COL2 (Abcam, MA, USA) or COL10 (Abcam) overnight at 4 °C. Following this incubation, the samples were treated with a secondary anti-rabbit polymer-horseradish peroxidase (HRP) antibody (Abcam) for 30 min. Staining was visualized using the DAB substrate and counterstained with hematoxylin. The stained areas within the chondrogenic pellets derived from infant ADSCs and UCSCs were quantified using image analysis software (Image Pro Plus 4.0, Media Cybernetics Inc., MD, USA). The obtained values were then compared to calculate the relative fold changes.

### 2.8 Osteogenic differentiation

Infant BMSCs and UCSCs, ranging from passages 3 to 5, were subjected to osteogenic differentiation using an osteogenic-induction medium (OIM). This medium, tailored for inducing osteogenic differentiation, consisted of DMEM supplemented with 10 % FBS, 50 μg/ml ascorbic acid-2 phosphate (Nacalai, Kyoto, Japan), 0.01 μM dexamethasone (Sigma-Aldrich), and 1 mM β-glycerol phosphate (Sigma-Aldrich). The cells were cultured under standard conditions at 37 °C with 5 % CO_2_ for a duration of 21 days.

### 2.9 Alizarine red S (ARS) staining

To assess osteogenic differentiation, both infant BMSCs and UCSCs were cultured in osteogenic differentiation medium for a period of 21 days. Subsequently, the cells were fixed and subjected to Alizarin Red S (ARS) staining, utilizing ARS obtained from Sigma-Aldrich. ARS, an anthraquinone dye, is commonly used to identify calcium deposits in cell cultures. Quantification of the osteogenesis induction in infant ADSCs and UCSCs involved extracting ARS from the stained cells using a 10 % cetylpyridinium chloride buffer (CPC). The optical density (OD) of the extract was measured at 550 nm using an ELISA reader (SpectraMAX 250; Molecular Devices, CA, USA) to quantify the extent of osteogenesis.

### 2.10 Tenogenic differentiation

Infant BMSCs and UCSCs, ranging from passages 3 to 5, were subjected to tenogenic differentiation using a tenogenic-induction medium (TIM). This specialized medium was prepared by supplementing Glutamax from Fisher Scientific (OH, USA) with 0.1 μg Primocin (InvivoGen, CA, USA), 50 μg/ml Ascorbic acid (AA; Sigma Aldrich), 50 ng/ml BMP-12, 100 ng/ml CTGF, and 10 ng/ml TGF-β3, all sourced from PeptroTech (London, UK). The cells were cultured under standard conditions at 37 °C with 5 % CO2 for a duration of 21 days to induce tenogenic differentiation [13].

### 2.11 Picro-Sirius Red staining

To assess the total collagen deposition in differentiated cells, these cells were seeded in 24-well plates (Corning life science) at a density of 3 × 10^3^ cells/cm^2^. Each sample was fixed using Bouin's solution (Bouin's Fixative, Electron Microscopy Sciences, PA, USA) for a duration of 1 h. Collagen fibers were stained using a solution of 0.1 % Picro-Sirius Red (ScyTek Laboratories) that was saturated in picric acid (Sigma-Aldrich). The collagen matrix deposition was then visualized under polarized light microscopy [14]. Quantification of the stained collagen was achieved by extracting Picro-Sirius Red from the stained cells using a 10 % CPC (cetylpyridinium chloride) buffer. The optical density (OD) of the resulting extract was measured at 550 nm using an ELISA reader (SpectraMAX 250; Molecular Devices, Sunnyvale, CA, USA). This measurement allowed for the quantification of collagen deposition.

### 2.12 Adipogenic differentiation

Infant BMSCs and UCSCs, ranging from passages 3 to 5, were subjected to adipogenic differentiation using an adipogenic-induction medium (AIM). This specialized medium consisted of DMEM supplemented with 10 % FBS, 50 μg/ml ascorbic acid-2 phosphate, 0.1 μM dexamethasone, 50 μM indomethacin (Sigma-Aldrich), 45 μM 3-isobutyl-1-methylxanthine (Sigma-Aldrich), and 1 μg/ml insulin (Sigma-Aldrich). These cells were cultured under standard conditions at 37 °C with 5 % CO2 for a duration of 21 days to induce adipogenic differentiation.

### 2.13 Oil Red O staining

Following differentiation, the cells were gently washed twice with phosphate-buffered saline (PBS). Subsequently, the cells were fixed with 10 % formalin for a period exceeding 1 h at room temperature. To visualize the extent of adipogenic differentiation, the cells were subjected to a 2-h staining with Oil Red-O (Sigma-Aldrich). To prepare the Oil Red-O working solution, a mixture of 15 ml of a stock solution (0.5 % in isopropanol) and 10 ml of distilled water was filtered through a PDVF membrane (0.2 μm) filter. Quantification of lipid accumulation was achieved by extracting Oil Red-O from the stained cells using isopropanol. The optical density (OD) of the resulting extract was measured at 510 nm using an ELISA reader (SpectraMAX 250). This measurement facilitated the quantification of lipid accumulation, thus serving as an indicator of the level of adipogenic differentiation.

### 2.14 Hepatogenic differentiation

UCSCs and infant ADSCs were subjected to a three-step induction process: Step 1 Induction (24 h): Cells were initially cultured in DMEM-LG containing 20 ng/ml human epidermal growth factor (hEGF, Sigma-Aldrich) and 10 ng/ml human fibroblast growth factor-basic (hBFGF, Sigma-Aldrich). This step was carried out at 37 °C in a 5 % CO2 environment for a duration of 24 h. Step 2 Induction (7 days): Following the first step, differentiation continued with a Step 2 Induction Medium, comprising DMEM-LG supplemented with 20 ng/ml human hepatocyte growth factor (hHGF, Sigma-Aldrich), 10 ng/ml hBFGF, and 0.61 mg/ml Nicotinamide (Sigma-Aldrich). This step extended for 7 days. Step 3 Induction (5–7 days): The final stage, Step 3 Induction, utilized DMEM-LG supplemented with 20 ng/ml Oncostatin (R&D System), 10ˆ-6 M Dexamethasone (Sigma-Aldrich), and ITS-premix from Corning life science. The cells were induced for 5–7 days as part of this step [15].

### 2.15 Immunofluorescence staining

To detect hepatogenic lineage differentiated cells on slides, primary antibodies against albumin from Taiclone Biotech Corp. (Taipei, Taiwan) were applied. Subsequently, the cells were incubated with DyLight 488-conjugated goat anti-mouse IgG secondary antibodies from Bethyl Laboratories Inc. (TX, USA). To highlight the cell nuclei, the slides were counterstained with DAPI for nuclear staining. The intensity of immunofluorescence was quantified using Image-Pro Plus software version 4.5.0.29. This allowed for the measurement and analysis of the fluorescence intensity to assess the presence of albumin and the degree of hepatogenic differentiation.

### 2.16 Statistical analysis

The quantitative analysis of the results was conducted using Prism software (version 5.03, GraphPad, CA, USA). The data were presented as mean values along with the standard error. To determine the statistical significance of the experimental results, a result was considered significant when the p-value was less than 0.05.

## 3 Results

### 3.1 The proliferation of infant BMSCs and UCSCs

**Fig. 1:**
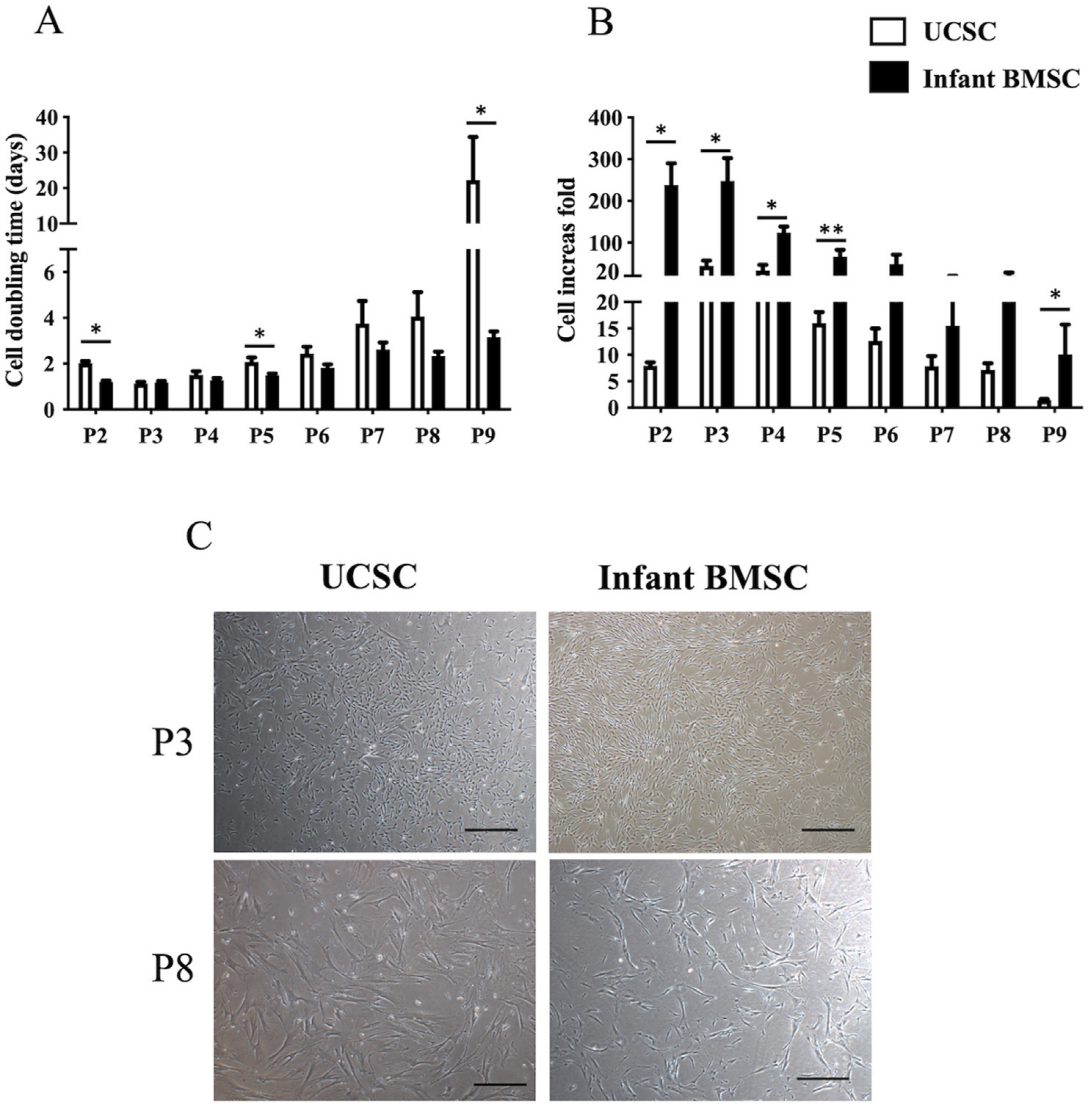
**Proliferation of the umbilical cord blood-derived mesenchymal stem cells (UCSCs) and infant bone marrow-derived mesenchymal stem cells (BMSCs)** (A) The fold increases in cell numbers and (B) doubling time periods of UCSCs and infant BMSCs were calculated and compared from passages 2 (P2) to 9 (P9). The data are presented as mean ± SEM, and multiple experimental replicates were conducted. Statistical significance for the comparisons between UCSCs and infant BMSCs at each passage was determined using the Mann-Whitney *U* test. The star symbol (∗) will indicate significant differences between the compared groups. The symbol "∗" denotes p < 0.05, and the symbol "∗∗" denotes p < 0.01. (C) Cell morphology of UCSCs and infant BMSCs at passages 3 (representing early passages) and 8 (representing late passages) was observed using a phase-contrast microscope (Nikon Eclipse TS100) with a magnification of × 40. The scale bar represents 100 μm.

A comparison of the proliferation between infant BMSCs and UCSCs was carried out by assessing cellular doubling times and the increase in cell numbers. In Fig. 1A, it's evident that the doubling time of infant BMSCs at passages 2, 5, and 9 was significantly lower than that of UCSCs. Furthermore, it illustrates that the fold increase in cell numbers for infant BMSCs from passages 2 to 5 and 9 was significantly higher than that of UCSCs (Fig. 1B). This suggests that infant BMSCs exhibit a more rapid rate of proliferation compared to UCSCs. The cell morphologies of infant BMSCs and UCSCs at passages 3 and 8 were imaged (Fig. 1C). The cell density of infant BMSCs was higher than that of UCSCs at both passages 3 and 8. The morphologies of infant BMSCs and UCSCs became flatter and more expanded at passage 8. The aforementioned results indicate that the preservation of proliferation potential in infant BMSCs is better than in UCSCs.

### 3.2 Comparison of senescence, replicative stress, and anti-oxidative ability were conducted between infant BMSCs and UCSCs

**Fig. 2:**
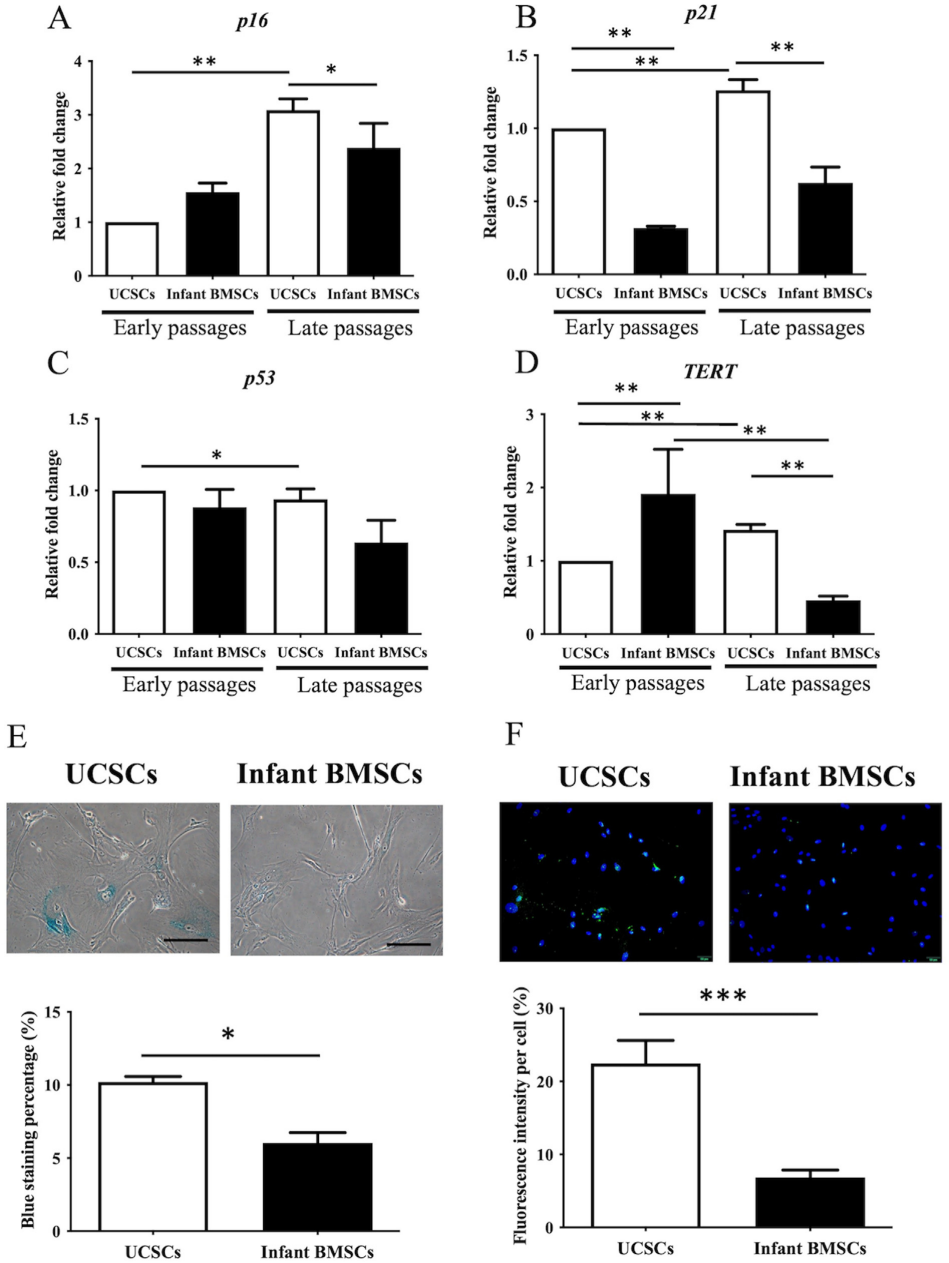
**Senescence and replicative stress in UCSCs and infant BMSCs** RT-qPCR analysis of senescence-related genes, including *p16* (A), *p21* (B), *p53* (C), and *TERT* (D) in UCSCs and infant ADSCs at passages 3–5 (early passages) and passage 7–9 (late passages). Each value was normalized to the expression of glyceraldehyde-3-phosphate dehydrogenase (*GAPDH*), and the relative fold changes of each value were compared to UCSCs at early passages. Using the data from UCSCs in the early passages as the baseline, we calculated the relative fold change of UCSCs in the late passages and infant BMSCs in both early and late passages. Statistical comparisons of differences were individually analyzed using Mann-Whitney U analysis for the following groups: differences between UCSCs in the early and late passages, differences between infant BMSCs in the early and late passages, differences between UCSCs and infant BMSCs in the early passage, and differences between UCSCs and infant BMSCs in the late passages. (E) Senescence-associated β-galactosidase (SA-β-gal) staining of UCSCs and infant BMSCs at late passages was detected using a Senescence Detection Kit, and images were captured using a Nikon Eclipse TS100 microscope. Blue-stained cells indicate positive SA-β-gal staining. The cell images were captured at 200 × magnification (the scale bar = 100 μm). (F) Immunostaining of γH2AX (green) and DAPI (blue) in UCSCs and infant BMSCs at late passages, detected using an Olympus BX43 microscope at a magnification of × 400, with the scale bar representing 100 μm. Quantification of γH2AX immunofluorescence intensity in the nuclei of UCSCs and infant BMSCs. Values, quantified using Image-Pro Plus v4.5.0.29, were normalized to the cell numbers and compared to show the relative fold changes. Mean ± SEM of quantified values with three replicates are expressed. Statistical significance of comparing UCSCs and infant BMSCs was determined by the Mann-Whitney *U* test. The star symbol (∗) will indicate significant differences between the compared groups. “∗" represents p < 0.05, "∗∗" represents p < 0.01, and "∗∗∗" represents p < 0.001.

In UCSCs, the expression levels of cyclin-dependent kinase inhibitor 2A (*p16*), cyclin-dependent kinase inhibitor 1A (*p21*), and telomerase reverse transcriptase (*TERT*) during late passages (passages 7 to 9) were significantly higher compared to the early passages (passages 3 to 5). However, the expression level of *p53* showed a reverse trend (Fig. 2A–D). In contrast, the expression of *TERT* in infant BMSCs during late passages was significantly downregulated compared to its levels during early passages. Additionally, *p21*, which plays a role in cell cycle regulation, was significantly downregulated in infant BMSCs at both early and late stages. Conversely, *TERT*, which is involved in maintaining genomic integrity, was significantly upregulated in infant BMSCs at early stages but downregulated at late stages (Fig. 2D). Senescence-associated beta-galactosidase (SA-β-gal) assays were used to evaluate cellular senescence, with senescent cells appearing blue (Fig. 2E). The percentage of SA-β-gal-positive UCSCs at late stages was significantly higher than that of infant BMSCs (Fig. 2E). To detect replicative stress, the presence of γH2AX, a marker, was examined in the nuclei of UCSCs and infant BMSCs at late passages, which appeared as green fluorescence (Fig. 2F). The quantified fluorescent intensities of infant BMSCs were significantly lower than those of UCSCs (Fig. 2F). These results suggest that infant BMSCs exhibit fewer signs of cellular senescence at late passages compared to UCSCs.

**Fig. 3:**
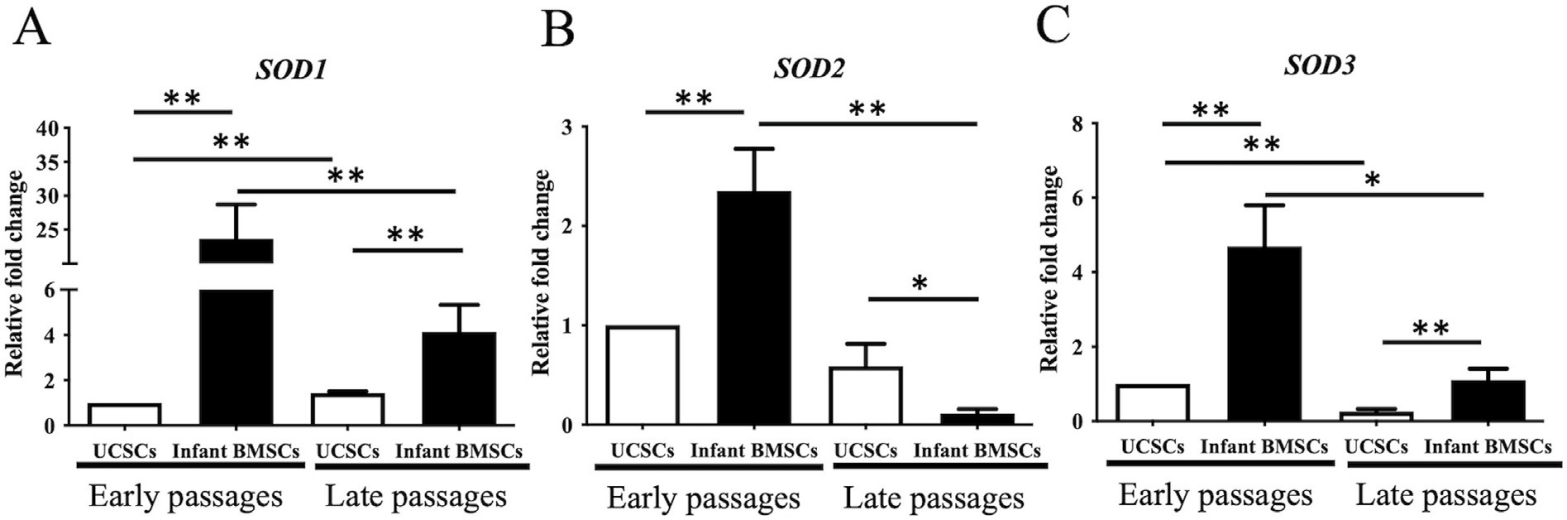
**Superoxide dismutases (SODs) expression levels in UCSCs and infant BMSCs** (A) The *SOD1*, (B) *SOD2*, and (C) *SOD3* genes of the UCSCs and infant BMSCs were analyzed by RT-qPCR, and the values were normalized to the expression of glyceraldehyde-3-phosphate dehydrogenase (*GAPDH*). Using the data from UCSCs in the early passages as the baseline, we calculated the relative fold change of UCSCs in the late passages and infant BMSCs in both early and late passages. Statistical differences were analyzed individually using the Mann-Whitney *U* test for the following groups: differences between UCSCs in the early and late passages, differences between infant BMSCs in the early and late passages, differences between UCSCs and infant BMSCs in the early passages, and differences between UCSCs and infant BMSCs in the late passages. Mean ± SEM of values with three experimental replicates are expressed. The star symbol (∗) will indicate significant differences between the compared groups. “∗" represents p < 0.05, and "∗∗" represents p < 0.01.

The expression of superoxide dismutase genes, specifically *SOD1*, *SOD2*, and *SOD3*, was assessed in both UCSCs and infant BMSCs at early and late passages. In infant BMSCs, the expression levels of *SOD1*, SOD2, and *SOD3* were significantly downregulated during the late passages in comparison to the early passages (Fig. 3A–C). Conversely, in UCSCs, *SOD1* exhibited significantly higher expression in late passages than in early passages (Fig. 3A), whereas *SOD3* was downregulated in late passages (Fig. 3C). When comparing the two cell types, it was observed that at early passages, the expression levels of all three *SODs* were significantly higher in infant BMSCs as compared to UCSCs (Fig. 3A–C). However, at late passages, the expressions of *SOD1* and *SOD3* in infant BMSCs were significantly higher than those in UCSCs, while the results for *SOD2* were the opposite.

### 3.3 Chondrogenic differentiation potential in the infant BMSCs and UCSCs

**Fig. 4:**
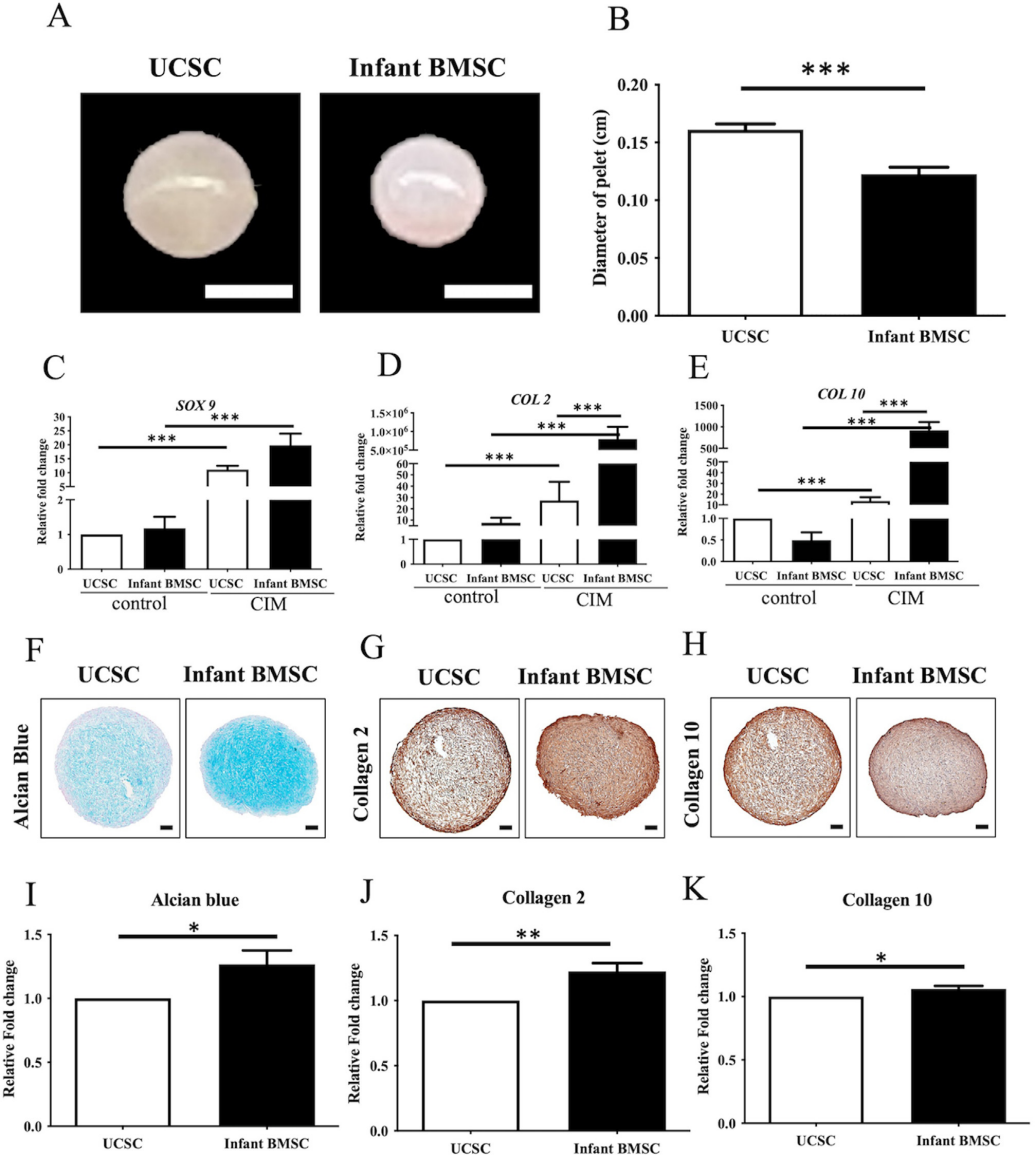
***In vitro*****chondrogenic differentiation of UCSCs and infant BMSCs** (A) Chondrogenic pellets differentiated from UCSCs and infant BMSCs at early passages (3x10^5^ cells/pellet) on day 21. (Scale bar = 1 mm) (B) Measurement of the diameters of the 21-day differentiated pellets. The expression levels of: (C) *SOX9* on day 7, (D) *COL2*, and (E) *COL10* on day 21 of the UCSC- and infant BMSC-differentiated pellets, detected by RT-qPCR. The values were normalized to the expression of glyceraldehyde-3-phosphate dehydrogenase (*GAPDH*). The gene expression of the undifferentiated cells was used as the control to compare with that of the UCSC- and infant BMSC-differentiated chondrocyte-like cells, and the relative fold changes were shown. Mean ± SEM of values with three experimental replicates were expressed. Statistical differences were analyzed individually using the Mann-Whitney *U* test for the following groups: differences between UCSCs in the control and chondrogenic factor-induced medium (CIM) groups, differences between infant BMSCs in the control and CIM groups, differences between UCSCs and infant BMSCs in the control groups, and differences between UCSCs and infant BMSCs in the CIM groups. (F) Pellets of the 21-day chondrogenic differentiated UCSCs and infant BMSCs were sectioned and stained with Alcian blue, (G) anti-collagen type 2 (COL2), and (H) anti-collagen type 10 (COL10) antibodies to detect the protein expression levels by immunostaining. The intensities of each staining were measured and normalized with the whole backgrounds of the sections. The value of the UCSCs was used as the standard to compare with that of the infant BMSCs, and the relative fold changes were shown. Mean ± SEM of values with three experimental replicates were expressed. Statistical significance of comparing the UCSC- and infant BMSC-differentiated pellets was determined by the Mann-Whitney *U* test. The star symbol (∗) will indicate significant differences between the compared groups. “∗” represents p < 0.05. “∗∗” represents p < 0.01. “∗∗∗” represents p < 0.001.

In comparison to UCSCs, infant BMSCs were able to differentiate into smaller chondrogenic pellets (Fig. 4A). The average diameter of the chondrogenic pellets differentiated from UCSCs on day 21 was approximately 0.15 cm, whereas that of infant BMSCs was about 0.13 cm, signifying a significant reduction in size compared to the UCSCs differentiated pellets (Fig. 4B). The expressions of *SOX9*, *COL2*, and *COL10* in both infant BMSCs and UCSCs differentiated chondrogenic pellets were significantly higher than in undifferentiated cells, which served as a control (Fig. 4C–E). When compared to UCSCs, the expressions of *COL2* (Fig. 4D) and *COL10* (Fig. 4E) were significantly higher in the chondrogenic pellets differentiated from infant BMSCs on day 21. The glycosaminoglycan (GAG) content of the chondrogenic pellets derived from infant BMSCs and UCSCs was stained with Alcian blue, and the protein expressions of COL2 and COL10 were analyzed through immunohistochemistry staining (Fig. 4F–H). The GAG intensities of the chondrogenic pellets generated from infant BMSCs and stained with Alcian blue were significantly higher than those of the UCSCs (Fig. 4I). Additionally, both COL2 and COL10 expressions in the pellets formed from UCSCs were significantly lower than those formed from infant BMSCs (Fig. 4J and K). Despite the smaller size of chondrogenic pellets derived from infant BMSCs in comparison to those from UCSCs, the results of gene expression and immunobiological staining indicated that the chondrogenic differentiation potential of infant BMSCs was superior to that of UCSCs.

### 3.4 Osteogenic differentiation potential in the infant BMSCs and UCSCs

**Fig. 5:**
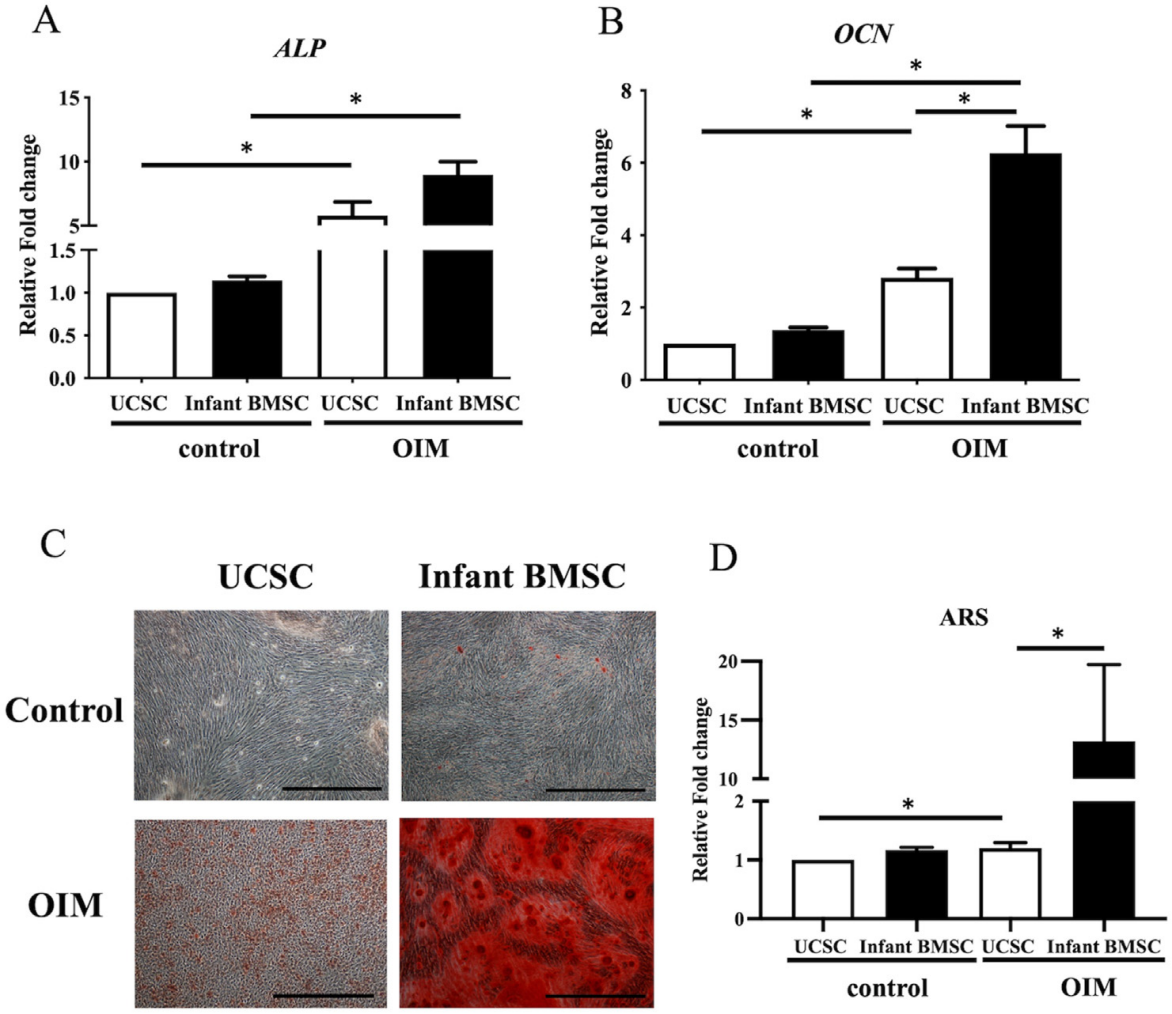
***In vitro*****osteogenic differentiation of UCSCs and infant BMSCs** The expression levels of osteocyte-related genes, including alkaline phosphatase (*ALP*) (A) and osteocalcin (*OCN*) (B) in UCSCs and infant BMSCs after treatment with the osteogenic factor-induced medium (OIM) for 21 days were detected using RT-qPCR. The expression values of each gene were normalized to the expression of glyceraldehyde-3-phosphate dehydrogenase (*GAPDH*). The gene expression of undifferentiated cells served as the control group for comparison with the UCSC- and infant ADSC-differentiated osteocyte-like cells, and the relative fold changes were presented. Statistical differences were analyzed using the Mann-Whitney *U* test for the following groups: differences between UCSCs in the control and OIM groups, differences between infant BMSCs in the control and OIM groups, differences between UCSCs and infant BMSCs in the control groups, and differences between UCSCs and infant BMSCs in the OIM groups. (C) Undifferentiated UCSCs and infant BMSCs were used as controls and stained with Alizarin Red S (ARS). The 21-day osteogenic differentiated UCSCs and infant BMSCs were confirmed with ARS staining and observed using a Nikon Eclipse TS100 microscope (magnification × 200, scale bar = 25 μm). (D) The OD of the ARS staining was measured at a wavelength of 550 nm. The OD of differentiated cells was normalized with that of controls, and the relative fold changes were presented. Mean ± SEM with three experimental replicates were expressed. Statistical significance in comparing the UCSC- and infant BMSC-differentiated osteogenic cells was determined using the Mann-Whitney *U* test. “ ∗" represented p < 0.05.

The relative expression fold change of osteogenic genes, such as alkaline phosphatase (*ALP*) (Fig. 5A) and osteocalcin (*OCN*) (Fig. 5B), in the osteogenically differentiated cells of infant BMSCs and UCSCs on day 21 were significantly higher than in undifferentiated cells. Moreover, the expression of *OCN* in the differentiated cells of infant BMSCs was significantly higher than in UCSCs' differentiated cells. To confirm osteogenic differentiation, calcium deposits in the osteogenically differentiated cells of both infant BMSCs and UCSCs on day 21 were stained with Alizarin Red S (ARS), displaying a distinct red color (Fig. 5C). The intensity of ARS staining in the differentiated cells of infant BMSCs was significantly higher than in those of UCSCs (Fig. 5D). These findings indicate that the osteogenic differentiation potential of infant BMSCs is superior to that of UCSCs.

### 3.5 Tenogenic differentiation potential in the infant BMSCs and UCSCs

**Fig. 6:**
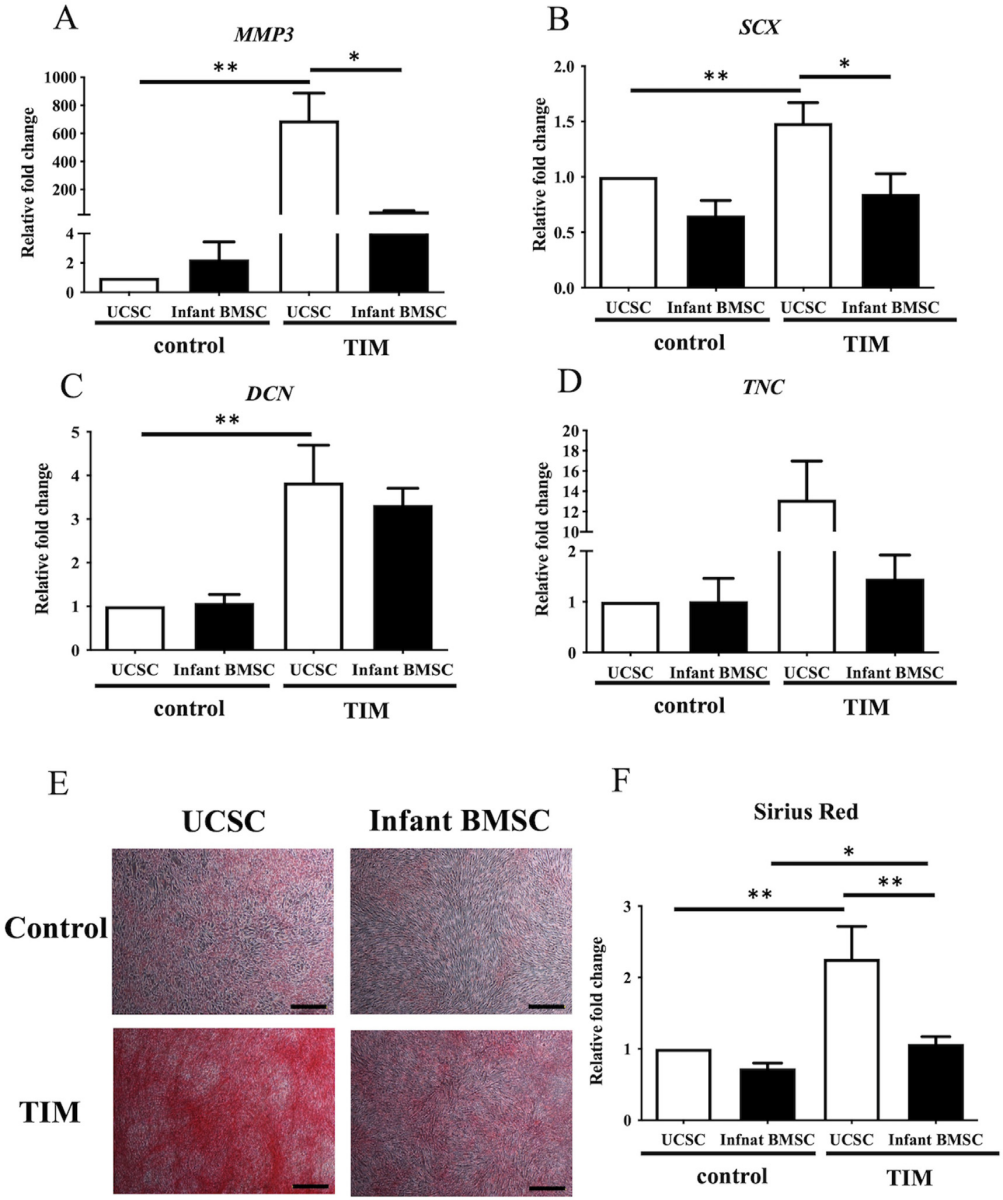
***In vitro*****tenogenic differentiation of UCSCs and infant BMSCs** The expression levels of tendon-related genes, including matrix metallopeptidase 3 (*MMP3*) (A), scleraxis (*SCX*) (B), decorin (*DCN*) (C), and tenascin-C (*TNC*) (D), in UCSCs and infant BMSCs after treatment with the tenogenic-induced medium (TIM) for 21 days, were detected using RT-qPCR. The expression values of each gene were normalized to the expression of glyceraldehyde-3-phosphate dehydrogenase (*GAPDH*). The gene expression of undifferentiated cells served as the standard for comparison with the UCSC- and infant BMSC-differentiated tendon-like cells, and the relative fold changes were presented. Statistical differences were analyzed using the Mann-Whitney *U* test for the following groups: differences between UCSCs in the control and TIM groups, differences between infant BMSCs in the control and TIM groups, differences between UCSCs and infant BMSCs in the control groups, and differences between UCSCs and infant BMSCs in the TIM groups. (D) Undifferentiated UCSCs and infant BMSCs were used as controls and stained with Picro-Sirius Red. The 21-day tenogenic differentiated UCSCs and infant BMSCs were confirmed with Picro-Sirius Red staining and observed using a Nikon Eclipse TS100 microscope (magnification × 40, scale bar = 100 μm). (E) The intensities of the Picro-Sirius Red staining were measured by detecting the OD at a wavelength of 550 nm and normalized to that of the controls. The relative fold changes were presented. Mean ± SEM with three experimental replicates were expressed. Statistical significance in comparing the UCSC- and infant BMSC-differentiated tenogenic cells was determined using the Mann-Whitney *U* test. “ ∗" represented p < 0.05. “ ∗∗" represented p < 0.01.

The expression of tendon-related genes, including matrix metallopeptidase 3 (*MMP3*) (Fig. 6A), scleraxis (*SCX*) (Fig. 6B), decorin (*DCN*) (Fig. 6C), and tenascin-C (*TNC*) (Fig. 6D), was compared between infant BMSCs and UCSCs after 21 days of tenogenic differentiation induction. The expression fold changes of *MMP3*, *SCX*, and *DCN* in the UCSCs' differentiated tenogenic cells were significantly higher than in the control cells. Conversely, the results for *MMP3* and *SCX* were significantly downregulated in the osteogenically differentiated cells of infant BMSCs. To confirm tenogenic differentiation, collagen in the 21-day differentiated tenogenic cells from both infant BMSCs and UCSCs was stained with Picro Sirius Red, displaying a red color (Fig. 6E). The measured intensities of Picro Sirius Red staining demonstrated that tenogenic differentiation in both UCSCs and infant BMSCs was significantly higher than in the control cells, with UCSCs differentiated cells exhibiting better results than those of infant BMSCs (Fig. 6F). The results indicate that the tenogenic differentiation potential of UCSCs may be higher than that of infant BMSCs.

### 3.6 Adipogenic differentiation potential in the infant BMSCs and UCSCs

**Fig. 7:**
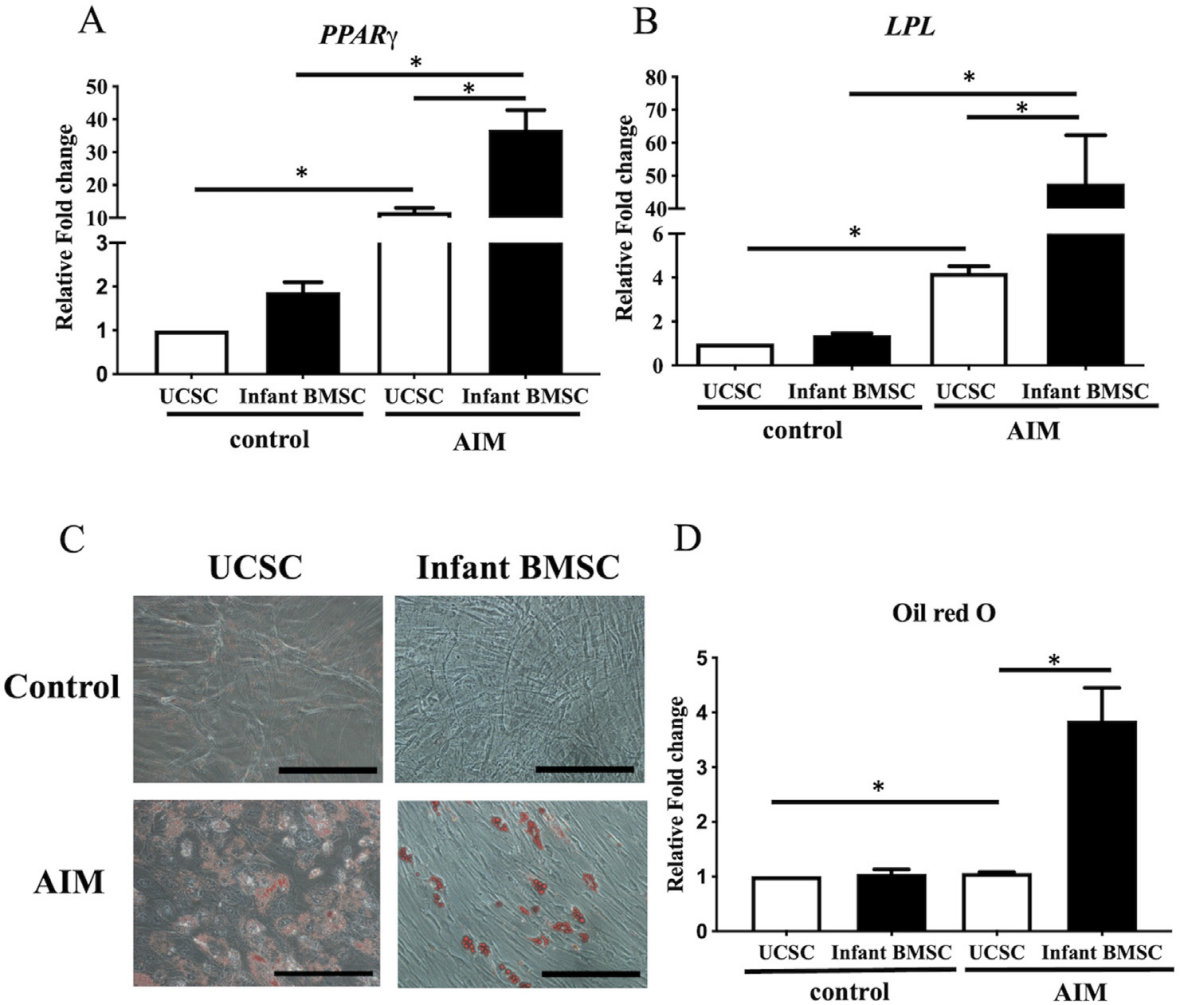
***In vitro*****adipogenic differentiation of UCSCs and infant BMSCs** The expression levels of adipose-related genes, including peroxisome proliferator-activated receptor-γ (*PPARγ*) (A) and lipoprotein lipase (*LPL*), in UCSCs and infant BMSCs after treatment with the adipogenic-induced medium (AIM) for 21 days were detected using RT-qPCR. The expression values of each gene were normalized to the expression of glyceraldehyde-3-phosphate dehydrogenase (*GAPDH*). The gene expression of undifferentiated cells was used as the standard for comparison with that of the UCSC- and infant BMSC-differentiated adipocyte-like cells, and the relative fold changes were presented. Statistical differences were analyzed using the Mann-Whitney *U* test for the following groups: differences between UCSCs in the control and AIM groups, differences between infant BMSCs in the control and AIM groups, differences between UCSCs and infant BMSCs in the control groups, and differences between UCSCs and infant BMSCs in the AIM groups. (C) Undifferentiated UCSCs and infant BMSCs were used as controls and stained with Oil Red O. The 21-day adipogenic differentiated UCSCs and infant BMSCs were confirmed with Oil Red O staining and observed using a Nikon Eclipse TS100 microscope (magnification × 200, scale bar = 25 μm). (D) The Oil Red O staining was extracted from the stained cells, and the optical density (OD) was measured at a wavelength of 510 nm. The OD of differentiated cells was normalized to that of undifferentiated cells, and the relative fold changes were presented. Mean ± SEM with three experimental replicates were expressed. Statistical significance in comparing the UCSC- and infant BMSC-differentiated adipogenic cells was determined using the Mann-Whitney *U* test. “ ∗" represented p < 0.05.

The fold changes in gene expressions of both peroxisome proliferator-activated receptor γ (*PPARγ*) (Fig. 7A) and lipoprotein lipase (*LPL*) (Fig. 7B) in adipogenically differentiated cells of infant BMSCs and UCSCs on day 21 were significantly higher than in control cells. Furthermore, the fold changes in gene expressions of *PPARγ* and *LPL* in the adipogenically differentiated cells of infant BMSCs were significantly higher than those in the UCSCs' differentiated cells. To confirm adipogenic differentiation, lipids in the adipogenically differentiated cells from both infant BMSCs and UCSCs on day 21 were stained with Oil Red O, displaying a red color (Fig. 7C). The intensities of Oil Red O staining in the differentiated cells of infant BMSCs showed a significant enhancement compared to that in the UCSCs differentiated cells (Fig. 7D). The potential to differentiate into the adipogenic lineage was higher in infant BMSCs compared to UCSCs.

### 3.7 Hepatogenic differentiation potential in the infant BMSCs and UCSCs

**Fig. 8:**
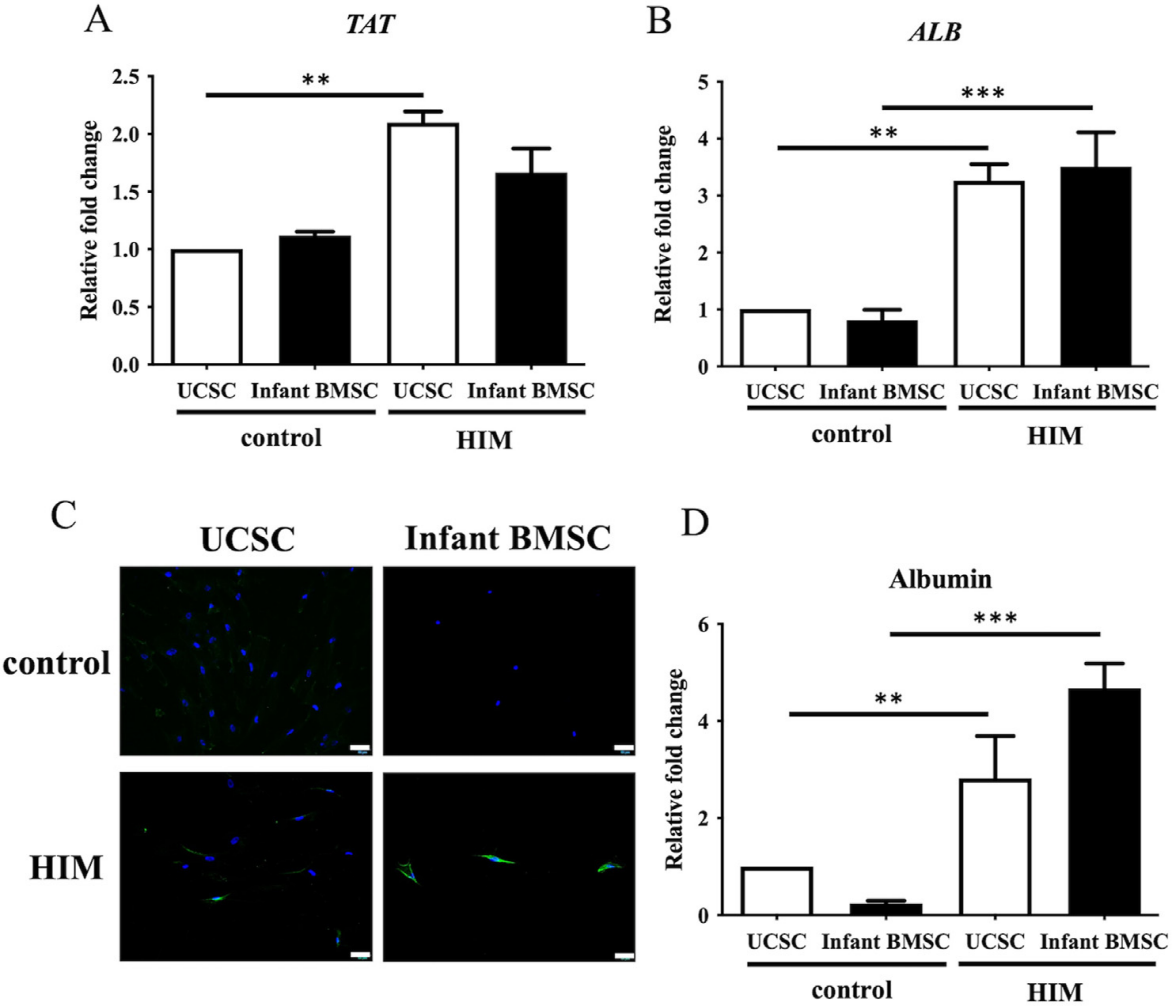
***In vitro*****hepatogenic differentiation of UCSCs and infant BMSCs** The expression levels of hepatocyte-related genes, including matrix albumin (*ALB*) (A) and tyrosine aminotransferase (*TAT*) (B), in UCSCs and infant BMSCs after treatment with the hepatogenic-induced medium (HIM) for 13–15 days were determined by RT-qPCR. The expression values of each gene were normalized to the expression of glyceraldehyde-3-phosphate dehydrogenase (*GAPDH*). The gene expression of undifferentiated cells was used as a standard for comparison with that of the UCSC- and infant BMSC-differentiated hepatocyte-like cells, and the relative fold changes were presented. Statistical differences were analyzed using the Mann-Whitney *U* test for the following groups: differences between UCSCs in the control and HIM groups, differences between infant BMSCs in the control and HIM groups, differences between UCSCs and infant BMSCs in the control groups, and differences between UCSCs and infant BMSCs in the HIM groups. (C) Undifferentiated UCSCs and infant BMSCs were used as controls and stained with DAPI and antibodies. Albumin protein in the UCSCs and infant BMSCs was detected by immunofluorescence staining, shown in green fluorescence, and evaluated with an Olympus BX43 microscope. The DNA stained by DAPI was shown in blue fluorescence (magnification × 400, scale bar = 100 μm). (D) Immunofluorescence intensity was quantified using Image-Pro Plus v4.5.0.29. Mean ± SEM with three experimental replicates were expressed. Statistical significance in comparing the UCSC- and infant BMSC-differentiated hepatocyte-like cells was determined using the Mann-Whitney *U* test. “ ∗∗" represented p < 0.01. “ ∗∗∗" represented p < 0.001.

The fold changes in expression of tyrosine aminotransferase (*TAT*) (Fig. 8A) and albumin (ALB) (Fig. 8B) in the hepatogenically differentiated cells of UCSCs were significantly higher than in the control cells. Additionally, ALB expression in the hepatogenically differentiated cells of infant BMSCs was significantly higher than in the control cells. However, both gene expressions were similar in the hepatogenically differentiated cells of infant BMSCs and UCSCs. Albumin expression was detected using immunofluorescence staining (Fig. 8C). Although the quantification of albumin expression in the hepatogenically differentiated cells of both infant BMSCs and UCSCs was significantly higher than in the control group, the comparison between infant BMSCs and UCSCs' differentiated cells was also similar (Fig. 8D). This indicates that the potential for hepatocyte differentiation is similar between the two cell types.

## 4 Discussion

Comparison between infant BMSCs and UCSCs revealed that infant BMSCs displayed higher proliferation rates and maintained better anti-senescence and antioxidation capabilities at late passages. Moreover, infant BMSCs exhibited an enhanced potential for chondrogenic, osteogenic, and adipogenic differentiation, while UCSCs showed a preference for tenogenic differentiation. In the context of hepatogenic differentiation, both cell types exhibited similar potential.

We conducted separate comparisons of proliferation between UCSCs and infant ADSCs, as well as UCSCs and infant BMSCs [16]. These experiments were carried out independently, and the data were not replicated. Interestingly, despite the separate studies, we observed that the growth curve for UCSCs was similar in both cases. In our previous study, we established that UCSCs were prone to senescence when compared to infant ADSCs [16]. However, our latest findings revealed that infant BMSCs displayed even higher proliferation rates than UCSCs, surpassing the levels observed in infant ADSCs. Both infant ADSCs and BMSCs were sourced from fat tissue and bone marrow extracted from phalanges of polydactyly patients without underlying organic diseases. These results highlight the potential of infant BMSCs as a promising material for tissue engineering applications.

Our previous study [16] highlighted that UCSCs exhibited lower anti-senescence and antioxidation properties. In comparison to infant ADSCs, UCSCs showed elevated expression of senescence-related genes, such as *p16*, *p21*, and *p53*, at early passages (P3-5). This indicated that the ability of infant ADSCs to delay senescence was superior to that of UCSCs. In the present study, the results revealed that the expressions of *p16* and *p21*, as well as the levels of β-gal expression and γH2AX immunofluorescence staining, in UCSCs at late passages were notably higher than those in infant BMSCs. This suggests that infant BMSCs possess strong anti-senescence capabilities, reducing the signs of senescence at late passages. Furthermore, in another study comparing senescence between dental pulp-derived MSCs (DPSC) and UCSCs at P6, as detected by β-gal analysis, it was observed that UCSCs exhibited significantly higher β-gal expression [17]. This finding further supports the notion that UCSCs are prone to senescence after multiple passages.

Previous studies have consistently demonstrated that infant ADSCs exhibit the highest gene expressions of *SODs* (*SOD1*, *SOD2,* and *SOD3*) when compared to adult ADSCs [18] and UCSCs [16]. Similarly, infant BMSCs also displayed elevated *SODs* gene expressions in comparison to adult BMSCs. Our current study reaffirms these findings, indicating that the expression of *SODs* in infant BMSCs was higher at early passages when compared to UCSCs. Moreover, the expression levels of *SOD1* and *SOD3* in infant BMSCs were also greater than those in UCSCs at the late passages. It is well-established that oxidative stress is a crucial factor contributing to stem cell senescence [19]. Collectively, the results from our series of studies suggest that SODs play a pivotal role in enabling infant mesenchymal stem cells to combat oxidative stress effectively.

In summary, the series of comparative studies reveals distinct differentiation potentials among different types of mesenchymal stem cells. When comparing infant ADSCs with adult ADSCs, infant ADSCs exhibit a superior capacity for chondrogenesis, adipogenesis, and neurogenesis, while displaying a reduced tendency for osteogenic and tenogenic differentiation [18]. On the other hand, UCSCs favor tenogenic differentiation, displaying limited potential for chondrogenic, osteogenic, adipogenic, or neurogenic lineages [16]. Additionally, the comparison of adult BMSCs with infant BMSCs suggests that infant BMSCs possess greater potential for chondrogenic, osteogenic, tenogenic, and hepatogenic differentiation, although they do not favor adipogenic differentiation [10]. In this study, we also observed UCSCs' preference for tenogenic differentiation compared to infant BMSCs. It is worth noting that the differentiation tendencies in stem cells are closely linked to their inherent mechanical properties, such as elasticity and viscosity. UCSCs exhibit higher stiffness, leading to a preference for osteogenic over adipogenic differentiation. Conversely, ADSCs demonstrate the opposite differentiation tendency [6,[20], [21], [22]]. However, when compared to infant ADSCs and BMSCs obtained from children with polydactyly, UCSCs appear to favor tenogenic differentiation exclusively. This suggests that infant BMSCs possess superior differentiation potential.

Our study has several limitations. Firstly, we utilized a limited number of UCSC cell lines, which may not fully represent the diversity within this cell population. Additionally, the lack of a time course analysis for differentiated gene expressions could potentially lead to the oversight of delayed differentiation in UCSCs. For instance, previous research has suggested that adipogenic differentiation in UCSCs might require an extended period [23]. Moreover, the differentiation potential of different MSCs under identical conditions, using the same differentiation induction media, can yield varying effects on infant BMSCs and UCSCs. However, the underlying mechanisms responsible for the potential differences between infant BMSCs and UCSCs have yet to be revealed. One potential mechanism that influences the differentiation potential of infant BMSCs and UCSCs is epigenetic regulation. Research shows that MSC differentiation into lineages like osteogenic and adipogenic is driven by DNA methylation and histone modifications [24]. Signaling pathways, such as Wnt signaling, also play a crucial role in MSC fate decisions [25]. Additionally, oxidative stress, antioxidant capacity, and MSC aging affect differentiation potential [26,27]. Growth factors and cytokines, like TGF-β [28], further regulate lineage differentiation and may contribute to differences in infant BMSCs and UCSCs differentiation. These factors should be considered when interpreting the results and may warrant further investigation in future studies. Based on MSC-based clinical trials, the choice of MSC derived from different resources for treating specific diseases remains elusive. Comprehensive meta-analyses will be required for evident judgment. Overall, the in vitro expansion and differentiation provide stemness evaluation of the differences between MSC derived from variable sources, and this can make foundations for deciding which MSC to use for specified clinical applications.

In comparison to UCSCs, infant BMSCs displayed superior proliferation abilities and better preservation of stemness. These findings underscore the potential value of BMSCs collected from children for tissue engineering applications.

## Authors’ contributions

Conception and design of study: Szu-Hsien Wu, Jin-Huei Yu, and Jung-Pan Wang. Acquisition of data and/or analysis and interpretation of data: Yu-Ting Liao, Po-Hsin Chou, Ming-Hsuan Wen, and Kuang-Kai Hsueh. And all authors participated in drafting the article or revising it critically for important intellectual content, and gave final approval of the version to be submitted and any revised version.

## Ethical statement

The protocol titles for human ethics are as follows: “Comparison and investigation of the chondrogenic differentiation potential between adult and infant bone marrow stem cells” and “Comparison and investigation of the chondrogenic differentiation potential and the role of superoxide dismutases (SODs) between adult and infant bone marrow mesenchymal stem cells.” The collection of human BMSCs was approved by the Institutional Review Board (IRB) of the Taipei Veterans General Hospital, with approval numbers 2019-08-004A and 2020-06-007B, respectively. These approvals were granted on December 17, 2019, and July 23, 2020, respectively. The bone tissue was obtained with informed consent from participants, or from their parent or legal guardian in the case of children under 16, who were included in the study.

## Funding

This study was supported in part by grants from the 10.13039/501100011912Taipei Veterans General Hospital (V111C-231), the Ministry of Science and Technology (MOST 111-2314-B-075-057-MY3), and the 10.13039/100015820Taoyuan General Hospital, Ministry of Health and Welfare (PTH111063).

## Declaration of Generative AI and AI-assisted technologies in the writing process

During the preparation of this work the author(s) used ChatGPT in order to enhance English editing. After using this tool/service, the author(s) reviewed and edited the content as needed and take(s) full responsibility for the content of the publication.

## Declaration of competing interest

The authors declare the following financial interests/personal relationships which may be considered as potential competing interests: Jung-Pan Wang reports financial support was provided by Taipei Veterans General Hospital. Szu-Hsien Wu reports financial support was provided by Ministry of Science and Technology. Jin-Huei Yu reports financial support was provided by Ministry of Health and Welfare Taoyuan General Hospital. If there are other authors, they declare that they have no known competing financial interests or personal relationships that could have appeared to influence the work reported in this paper.
