# Supplementary material for: Comparison of infant bone marrow- and umbilical cord-derived mesenchymal stem cells in multilineage differentiation
Source: Regen Ther. 2024 Oct 3;26:837–49. doi: 10.1016/j.reth.2024.09.011 (PMC11488484; doi:10.1016/j.reth.2024.09.011)
Supplement: Multimedia component 1 [file mmc1.pptx]

## Slide 1
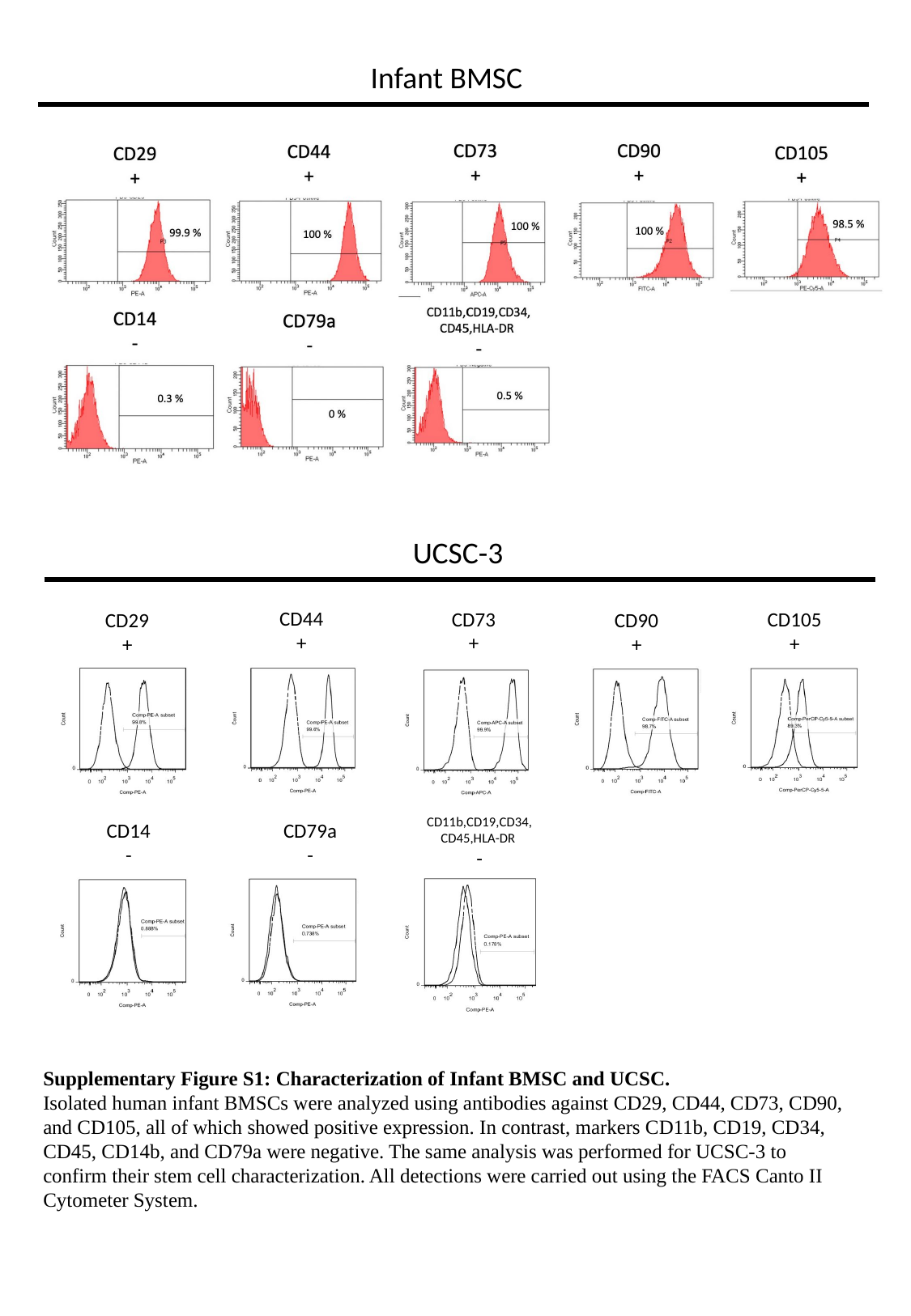

Infant BMSC
UCSC-3
CD44
+
CD73
+
CD105
+
CD90
+
CD29
+
CD11b,CD19,CD34,
CD45,HLA-DR
-
CD79a
-
CD14
-
Supplementary Figure S1: Characterization of Infant BMSC and UCSC.
Isolated human infant BMSCs were analyzed using antibodies against CD29, CD44, CD73, CD90, and CD105, all of which showed positive expression. In contrast, markers CD11b, CD19, CD34, CD45, CD14b, and CD79a were negative. The same analysis was performed for UCSC-3 to confirm their stem cell characterization. All detections were carried out using the FACS Canto II Cytometer System.
